# Ship Fever at the Bellevue Hospital

**Published:** 1847-10

**Authors:** D. M. Reese, C. A. Lee

**Affiliations:** Resident Physician


### RECORD OF MEDICAL SCIENCE.

### *Ship Fever at the Bellevue Hospital.*

To the Editor of the New York Journal of Medicine:

*Dear Sir:*—In compliance with your request, I send you the enclosed brief statement of my observations and experience at Bellevue Hospital, during the late prevalence of Ship Fever, in the midst of which I was appointed to the charge of the establishment, in May last.

Your readers are doubtless informed of the immense influx of pauper immigration into the port of New York, especially from Ireland, during the present year. Their impoverished condition by reason of the famine at home, had superinduced a morbid *predisposition*, which only needed an *exciting cause* to develope those functional disturbances, which characterize fever. Their circumstances on ship-board, being crowded together between decks, to an extent little short of that said to be resorted to in slavers; short of provisions, and even of water; without the possibility of cleanliness or ventilation; presented a combination of morbid agencies which could scarcely fail to generate not merely fever, but *pestilential fever*, in some malignant form. Amid such accumulated filth and wretchedness, it is not at all wonderful that such frightful reports of disease and death should herald almost every arrival; nor could it reasonably be expected otherwise, than that our hospitals and almshouses should be crowded with the sufferers, borne thither on landing, either already sick, or so deeply infected as to render escape from an attack of fever scarcely possible.

Such has accordingly been the fact, and to an extent which may be estimated from the following statistical items prepared from the best data found in the hospital.

From the 1st of January, 1847, to May 25th, this hospital was under the charge of my predecessor in office, who admitted during this period 769 cases, many of them direct from ship-board. Of these, as appears by the record, 306 were discharged cured—154 died, and 309 remained in the hospital under treatment, a large portion of whom were nearly moribund, when I assumed the charge as resident physician.

At that time, May 26th, 1847, there were over 800 patients in the house, 309 were suffering with ship-fever. Cases of the latter description were then admitted at the rate of 60 to 80 per day, so that within a week or two, notwithstanding deaths and discharges, the number had increased to 1147 in the hospital, over 600 of whom were ill with the ship-fever.

With this large number, being so much beyond the capacity of the hospital buildings to accommodate, and alike beyond the available resources of the establishment, to furnish immediate supplies, very great suffering, and appalling mortality were unavoidable, until other and more extended facilities could be provided. The crowded state of the wards, which could not be adequately ventilated or even cleansed, had already resulted in developing an *endemic* atmosphere, for only in confined and impure air is this fever ever infectious. The proof that it had become so, was seen in the fact that a majority of the medical assistants had already sickened, and one of them had died. Many of the nurses were sick and some of them dead. Moreover, the adjacent alms-house building, with a population of 1500, began to give proof of having become infected, no less than 17 cases of the identical form of fever having occurred within 48 hours, some of which were rapidly fatal.

Under these appalling circumstances, 80 tents were pitched upon the adjacent green, and were immediately filled with the patients from crowded wards, which thus admitted of being whitewashed, cleansed and ventilated. A number of shanties in the yard were soon filled in like manner and for the same purpose. By these and the like extemporaneous devices, ample room was provided for the increasing number of patients, and a thorough purification of the apartments was attained. And here, as in other cases, it was soon demonstrated, that the patients who were removed into the open air immediately improved, and a very large portion of them soon recovered. The new cases were very generally placed in the tents, and my records show that over 200 cases were discharged cured from the tents, no one of whom had entered the walls of the hospital.

But to return to the statistics of the hospital. It appeared by the books that from May 25th to August 3d, 1847, there were admitted into the hospital 917 cases of ship-fever, which, added to those remaining in the house, makes the aggregate of cases treated within that period amount to 1226 during about ten weeks.

By a table, prepared August 3d, the following results are furnished, viz.:

| | |
|---|---|
| Discharged cured, | 724 |
| Died, | 193 |
| Convalescent, | 129 |
| Remaining under treatment, | 180 |
| Whole number as above, | 1226 |

From these respective data, the whole number of cases of ship-fever admitted into this hospital since Jan. 1st, 1847, has been 1995, of whom 347 have died, which latter number gives the aggregate mortality, down to Aug. 3d, the date to which the calculations have been made. It gives an appalling aspect to the fever, but furnishes no just criterion of the necessary fatality of the disease, nor of its want of amenability to judicious treatment; as appears from the fact that a very large proportion of the cases were brought in suffering under the profound coma which characterizes late periods of the disease, and still worse, very many were moribund when they reached the hospital. No less than 17 died during one week, within four hours after admission, while four, during the same week, were dead before they could be carried to their beds. The folly and inhumanity of sending dying persons in a heavy carriage over the rough pavements, to so distant a hospital, must be painfully obvious, extinguishing, as it often does, the spark of life which remains, and which else might possibly be revivified.

The following extracts from the weekly reports since May 26th, 1847, though they include deaths from *all diseases*, may aid in arriving at the comparative mortality, before and after the cleansing and ventilation of the hospital, and the removal of hundreds of the sick for treatment into the open air; a measure resorted to in this instance from necessity, but found highly salutary and useful, especially in the management of ship-fever.

| | No. of patients. | Deaths. |
|---|---|---|
| 1st week, | 1142 | 71 |
| 2d " | 1020 | 59 |
| 3d " | 956 | 35 |
| 4th " | 985 | 39 |
| 5th " | 981 | 41 |
| 6th " | 956 | 38 |
| 7th " | 937 | 30 |
| 8th " | 947 | 31 |
| 9th " | 868 | 25 |

During this period, the cases of ship-fever numbered about one half of all the diseases in the house; and differing but little from this ratio in the proportion of deaths, showing that the mortality, of late, is not greater than that resulting from other diseases. Since Aug. 3d, the cases of fever have been diminishing rapidly, and the whole mortality of the hospital has not exceeded 18 weekly. At present, Aug. 23, there are not more than 50 cases of the fever in the establishment, the most of these having been landed at Quebec, whence they have found their way to this city, and these less malignant and dangerous than those we have heretofore treated.

This subsidence of the fever, so that the discharges now exceed the admissions, has reduced the aggregate of patients below 700, so that the tents have now been emptied, and I find ample room for all in the wards of the hospital; while the comfortable state of these wards, and the encouraging condition of the sick, are sources of no small gratification.

In respect to the peculiar nature and specific character of this fever, the late period at which the patients generally reached the hospital, has precluded very accurate observations. Occasional opportunities, however, have occurred for watching its inception and progress in the persons who sickened on our premises. Many of these came hither from on ship-board apparently in health, but really in a state of morbid predisposition, though latent, which soon after developed itself in an attack of the fever, as well characterized as were the cases which had fully developed the disease on board of the same ships. While others occurred among the assistant physicians, orderlies and nurses in the hospital, which were as well marked by pathognomic signs throughout their whole course; nor could they be discriminated in their symptoms, stages, or duration, from those direct from the ships. In the latter examples it is evident that the malady originated from the pestilential atmosphere generated within our walls, which was sufficiently potent at one time to become both the remote and the exciting cause, the same identical agency being sufficient to produce the predisposition, and afterwards develope the fever.

Nor is there the slightest foundation for the suspicion of any specific contagion in the case, as is seen in the fact that no new instances of the fever have occurred in the premises, since the cleansing, purification, and ventilation of the establishment has been effected; so that the rationale of the infection which was undoubtedly *endemic* here, and had become *epidemic* in the vicinity, must be obvious; and is precisely the same as that existing on ship-board in the instances of sickness and death which have become so lamentably notorious. The want of pure air, of wholesome food, and of pure water, are privations which by a physical necessity generate disease. In a crowded hospital, as well as in a crowded ship, filthy apartments, ill-ventilated wards, and the confined air resulting from such untoward circumstances, have from time immemorial been known to be such violations of hygenic laws, as will develope pestilential fever. All who inhale such an atmosphere for any length of time become sick, and each sufferer by the morbid exhalations from his skin and lungs, contributes to augment the infection, and increase the sources of danger to himself and others. Still worse, if amid such a crowd there be, as is too often the case, a neglect of personal cleanliness, and a failure to remove the morbid excretions, which, if allowed to remain, vastly add to the intensity of the atmospheric poison. Such are the precise circumstances under which, in certain latitudes and given temperatures, *ship*-fever, jail-fever, and *hospital*-fever have been generated and perpetuated. Such a pestilence may be manufactured to order; and may be arrested with equal facility by obeying the laws of hygeine, instead of violating them. So much for the contagiousness of the ship-fever. As to the figment of "contingent contagion," it is only contingent nonsense.

Those who have imagined that in this much dreaded "ship-fever," there has been any distinctive or specific character constituting it a new disease, or in any sense *sui generis*, must have had very limited opportunities for observing it. Nor is there anything in its pathology or treatment contradistinguishing it from the family of congestive fevers, of which it is a familiar variety, modified, however, by different causes, but always characterized by the same type. Indeed, this identical fever has been annually observed to greater or less extent on board ships, in our hospitals and in other crowded apartments of our city, inhabited by a degraded population; and it has often appeared in jails, prisons, etc., in various sections of our country.

The symptoms, course and type of the ship-fever here, have been identical with those of that form of malignant disease denominated "Typhus Petechialis," modified in different examples by age, sex, temperament, habits of living, etc., but all bearing the impress of the same cause, proving their absolute identity in nature, though differing in degree. Always congestive, often inflammatory, and very frequently both the one and the other, constituting the mixed fever of modern writers.

In the example which came under our observation sufficiently early to allow of recording and discriminating its premonitions and development, there was found very great uniformity. The earliest and most prominent symptoms of an attack, have been sudden loss of strength, soon followed by a sense of overwhelming debility, while as yet there was no appreciable functional disturbance. A disinclination for food and an inability to sleep supervened, often with great rapidity. The tongue and eyes usually presented the first distinct morbid appearances, the former becoming coated and the latter red and watery, while no manifest febrile symptoms, properly so called, were discoverable. At this period a slight chilliness was often complained of, though not always cognizable. A full chill did occasionally occur, but it was but very seldom. Nausea was very often present, and in a few rare cases vomiting, with or without diarrhœa, seemed to designate the development of the fever. The skin remained dry, and slightly elevated in temperature, while the pulse indicated the presence of indirect debility, though differing in frequency very little from the natural standard for several days, when it usually became accelerated somewhat, and in bad cases soon fell below 70 in the minute.

The most constant characteristics of this fever were great apathy, and apparent indifference to life; sudden and continued deafness; a mental torpor which could not be roused to sensibility, and the absence of all pain, or at least of all complaining, except of weakness, which was universally present throughout the whole course of the disease. Petechiæ, though not invariably present, were very generally so, occasionally over the entire body, but more frequently upon the neck, chest, and abdomen, in which situations they usually were most visible and most numerous. In general, these appeared about the seventh day, but often earlier, sometimes on the third day; and I have seen them in great numbers as late as the seventeenth day of the disease, with and without sudamina, especially over the abdomen. The tongue presented very variable appearances, sometimes continuing white and thickly coated throughout, more frequently, however, becoming dry, brown, and even black, with occasional sordes, but the latter very rare, even in fatal cases. Delirium was very generally present after the seventh day, followed after a few days by coma, stertorous breathing, and subsultus tendinum, but these latter symptoms were not frequent.

In most of the cases, prior to the occurrence of delirium, a diarrhœa to greater or less extent was present, which was difficult to control by the usual remedies, when the excretions were biliary in their character; while it readily yielded under other circumstances, to a single dose of castor oil and laudanum. When this diarrhœa ceased, either spontaneously or as the result of medication, if at the same time the skin was open, convalescence was usually rapid, and a favourable prognosis might be safely made.

Relapses were frequent, even after entire convalescence, but most generally the result of some error in diet. In these the worst cases were those in which diarrhœa recurred, or erysipelatous inflammation exhibited itself. The latter cases were numerous, and sometimes fatal. Extensive suppurations of the parotid and other glands were among the worst sequelæ, and these mischiefs were observed especially with the intemperate, who were so numerous as to greatly augment the mortality of the hospital.

In respect to the treatment of this fever, there could be but very little difference of opinion among practical men, in reference to the majority of those patients received into this hospital, arriving, as they very generally did, in the later periods of the disease. So plainly was blood-letting contra-indicated, that, with the exception of a single instance, it was never resorted to by myself or assistants, nor was it even proposed by any of my medical friends who visited us, after inspecting the patients. Cupping was used in a few examples for the relief of local complications, and then always with advantage. But in general the aspect of the case forbade any depletory measures, or other active treatment. Even a single drastic purge was inadmissible, nor could it be given with impunity.

The successful means adopted by us may be thus detailed. A mild laxative was prescribed, if necessary, in the beginning, after which a dose of the Sp. Mindereri, with or without a grain of Ipecacuana, was given every hour or two, according to the urgency of symptoms, and this course steadily pursued until free perspiration was induced. Meanwhile nutritious drinks were used, such as oat-meal gruel, rice or barley water, arrow root and milk, beef tea, and the like. Ice and iced water were freely used, and when much heat was present, the head, neck, and body were sponged with ice water. Mustard plasters and blisters were used when diarrhœa or delirium supervened, or any indications were present of local lesions, or increased debility. So soon as any flagging of the pulse appeared, milk punch, wine whey, or brandy and ammonia were resorted to, and continued so long as stimulation was called for. These latter agencies had to be used to a very great extent in many cases, and with the best results.

Such was the general course of treatment, modified as circumstances demanded. When the diarrhœa became threatening, injections of nitrate of silver, a drachm to the pint, were found of great value. Dysenteric symptoms were arrested by calomel, opium, and ipecacuana, with injections of iced water. Erysipelatous complications were treated chiefly with quinine, and mercurial ointment, and occasionally by blisters and nitrate of silver. And so of local lesions, which but rarely occurred, the principles of rational medicine being our guide. The limits assigned me forbid greater particularity or amplification.

The pathological results of this fever, as shown by dissection, will soon be given to the profession in the forthcoming report of the Committee of the New York Academy of Medicine, from the notes of our excellent friend, Dr. Sabine, who, as one of the committee, has been pursuing that branch of inquiry. I may be pardoned, therefore, for the present, in only indicating what will then appear, viz.: that effusion in the ventricles of the brain was discoverable in almost every fatal case, and this for weeks together, while the autopsies were daily made. The absence of the intestinal ulcerations, which characterize the Dothinenteritis of the French writers, proved that our endemic, at least, has been called Typhoid fever by a misnomer, while the cerebral mischiefs so constantly observed, identify this fever with the other varieties of Typhus fever, being the petechial species of the genus; its malignity and danger having been the result of causes already indicated as accompanying its origin and prevalence.

Should this desultory letter serve your present purpose, written as it has been amid pressing avocations here, you may shortly hear from me again on the same topic, when more leisure shall be allowed me. For any measure of success which has accompanied my exertions in this hospital, I am greatly indebted to the diligence and toil of my assistants, Drs. O'Neil, Wendel, Mott, and Davis, who, with myself, providentially escaped the disease, while all the rest were visited by one or more attacks of fever. I am likewise under many obligations to Dr. F. Campbell Stewart, and Dr. A. V. Stout, who, at the most critical period, volunteered their valuable services and spent weeks with me in the hospital. Many others of my professional brethren, by their frequent visits, their counsel and sympathy in the trying duties to which I have been called, might be worthily mentioned, for all are gratefully remembered. Happily, none of my medical assistants fell victims to the fever, though eight of them were sick, and some of them suffered severe and dangerous attacks. In their treatment, Drs. Chalmers, Cheesman, Johnson, and others of the profession, rendered unremitting and valuable aid. You will forgive me for thus alluding incidentally and gratefully to these "friends in need," though writing for a Medical Journal, for I should do violence to the impulses of my heart, should I refrain. Their kind offices merit a better memorial, and serve to make us prouder of our noble profession. With great respect, I remain,

Your obedient servant,

D. M. REESE, Resident Physician.

Prof. C. A. Lee, M. D.

*Aug.* 28th, 1847.

